# A Brief Training Module Improves Recognition of Echocardiographic Wall-Motion Abnormalities by Emergency Medicine Physicians

**DOI:** 10.1155/2011/483242

**Published:** 2011-07-02

**Authors:** Chris Kerwin, Laura Tommaso, Erik Kulstad

**Affiliations:** Department of Emergency Medicine, Advocate Christ Medical Center, 4440 West 95th Street, Oak Lawn, IL 60453, USA

## Abstract

*Objective*. Wall-motion abnormality on echocardiogram is more sensitive in detecting cardiac ischemia than the electrocardiogram, but the use of bedside echocardiography by emergency physicians (EPs) for this purpose does not appear to be widespread, apparently due to limited data on proficiency of EPs for this task. We sought to determine the effect of a brief training module on the ability of EPs to recognize wall motion abnormalities on echocardiograms. *Methods*. We developed a brief training and testing module and presented it to EPs. After baseline testing of 15 echocardiograms, we presented the 30-minute training module, and administered a new test of 15 different echocardiograms. Physicians were asked to interpret the wall motion as normal or abnormal. *Results*. 35 EPs over two separate sessions showed significant improvement recognition of wall-motion abnormalities after the brief training module. Median score on the baseline test was 67%, interquartile range (IQR) 53% to 80%, while the median score on the posttraining test was 87%, IQR 80% to 87%, *P* < .001, independent of time in practice or prior training. *Conclusion*. With only brief training on how to recognize wall motion abnormalities on echocardiograms, EPs showed significant improvement in ability to identify wall motion abnormalities.

## 1. Introduction

Echocardiography is used frequently in the evaluation of patients with chest pain for the determination of wall-motion abnormalities indicating cardiac ischemia or infarction. Echocardiography has been shown to be more sensitive in detecting cardiac ischemia than the electrocardiogram (EKG), which is a more definitive tool for infarction only [1]. The sensitivity of echocardiography for predicting cardiac events within 4 hours of presentation was found to be 91%, compared with 40% for the ECG [2]. 

Prior experimental and echocardiographic studies during coronary angioplasty demonstrate that regional asynergy appears before electrocardiographic changes or pain [3]. 

The use of echocardiography in the emergency department (ED) has historically been emphasized in diagnosis and treatment of pericardial effusion and in confirmation of cardiac standstill [4, 5]. For emergency physicians to evaluate for the presence of regional wall-motion abnormalities is far less common even though this procedure has been proven to be a useful diagnostic tool for acute ischemia [3, 6]. The sensitivity of echocardiography alone for diagnosing myocardial infarction was found to be 91 percent [7].

Studies have demonstrated the ability of EPs to interpret echocardiograms at an acceptable accuracy rate for left ventricular systolic dysfunction [8], cardiac standstill [9], nontraumatic cardiac rupture [10], and pericardial effusions [5, 11], among other pathologies [12, 13], but limited data exist on the ability of emergency physicians to interpret regional ischemic wall motion abnormalities. 

Since diagnosis of myocardial ischemia via echocardiogram is optimized in the setting of active chest pain, it is a relevant procedure to be utilized in the ED. Obtaining an echocardiogram in the traditional fashion with a sonographic technician and interpretation by a cardiologist is too time consuming to be warranted for the acute nature of myocardial ischemia.

Whether EPs can develop the adeptness to accurately diagnose regional ischemic wall-motion abnormalities is uncertain, especially given they have historically had lesser exposure to echocardiographic images compared to a cardiologist. However, given that ED echocardiography is becoming more utilized and cardiac patients are in no short demand, they can potentially gain a great deal of exposure. 

We sought to determine the effect of a brief training module on the ability of EPs to recognize wall-motion abnormalities on echocardiograms in order to preliminarily assess whether EPs can eventually be accountable for this skill, as they are for other ultrasonographic skills [14]. We hypothesized that use of the training module would significantly improve the ability of EPs to identify wall-motion abnormalities on echocardiographic images, therefore predicting eventual accountability. 

## 2. Materials and Methods

We performed a before and after analysis of physician interpretation of wall-motion abnormalities to evaluate the effect of a brief training module. The study was performed at our community hospital with tertiary cardiac care, which has 700 beds, an ED with 85,000 patients treated per year, and an emergency medicine residency-training program in a PGY 1–3 format. Eligible subjects for our study included emergency medicine residents and attendings willing to participate in the study. The study was approved by our hospital institutional review board. 

We developed a brief training and testing module using echocardiographic images from multiple sources available on the web and through training compilations. The training module consisted of demonstrations of echocardiographic windows along with clips of echocardiograms showing both normal and abnormal wall motion. The length of the module was designed to be presented in a total of 30 minutes. 

We established baseline performance by testing physician's interpretation of 15 echocardiograms prior to the presentation of the training module. We then administered a second exam of 15 different echocardiograms after the 30-minute training module. For each echocardiogram, EPs were asked to interpret the wall motion as normal or abnormal on a scoring sheet, which also asked for specific information on demographics (resident versus attending and year of training if in residency), self-described comfort with ultrasound, self-reported frequency of use of ultrasound, and whether any prior formal ultrasound training had been received.

We performed a repeated-measures analysis of test scores using a Wilcoxon Signed-Rank test to assess for each subjects' change in score after completion of the training module. We then evaluated the association between scores obtained and training status, prior completion of an ultrasound course, or self-reported comfort and frequency of ultrasound use. Analyses were performed with SPSS version 15 (SPSS Inc., Chicago, Ill, USA).

## 3. Results

**Figure 1:**
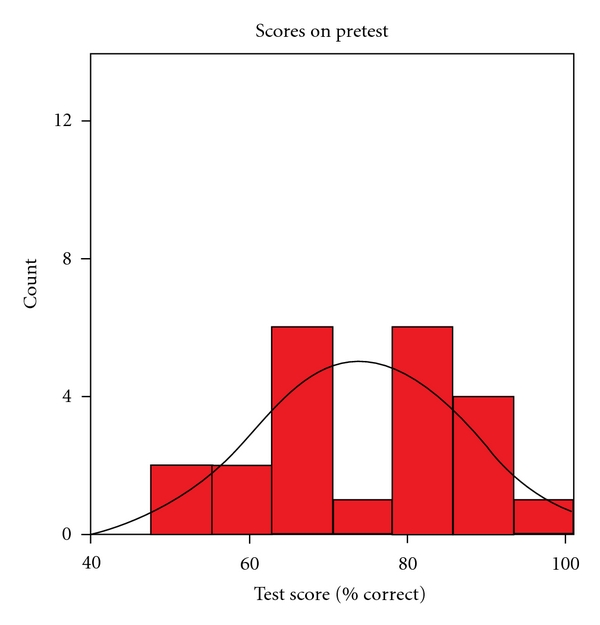
Pretest scores histogram.

A total of 35 subjects were evaluated in two separate sessions in the 2006-2007 academic year. The median score on the pretest was 67%, with an interquartile range of 53% to 80%.  Figure 1 shows the histogram of pretest scores. 

Test scores significantly improved after the training, with a median score on the posttest of 87%, and an interquartile range of 80% to 87%, (*P* < 0.001). 

**Figure 2:**
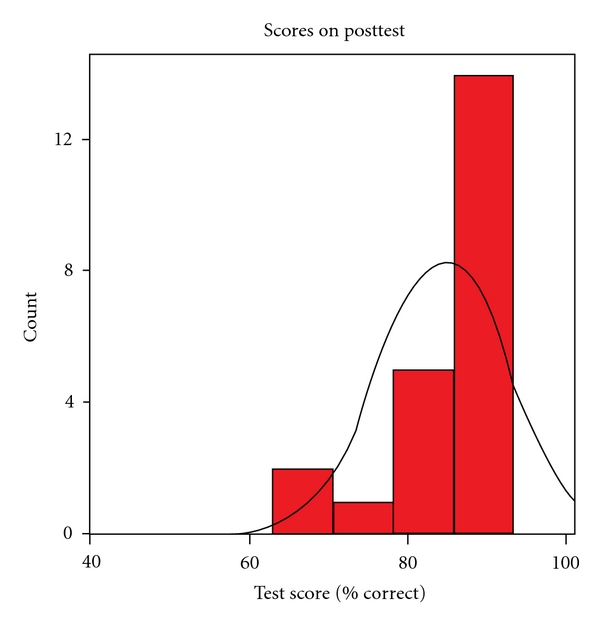
Posttest histogram.

Figure 2 shows the histogram of posttest scores.

 Multivariate regression showed no dependence on test score improvement with training status, prior completion of an ultrasound course, or self-reported comfort and frequency of ultrasound use. 

**Figure 3:**
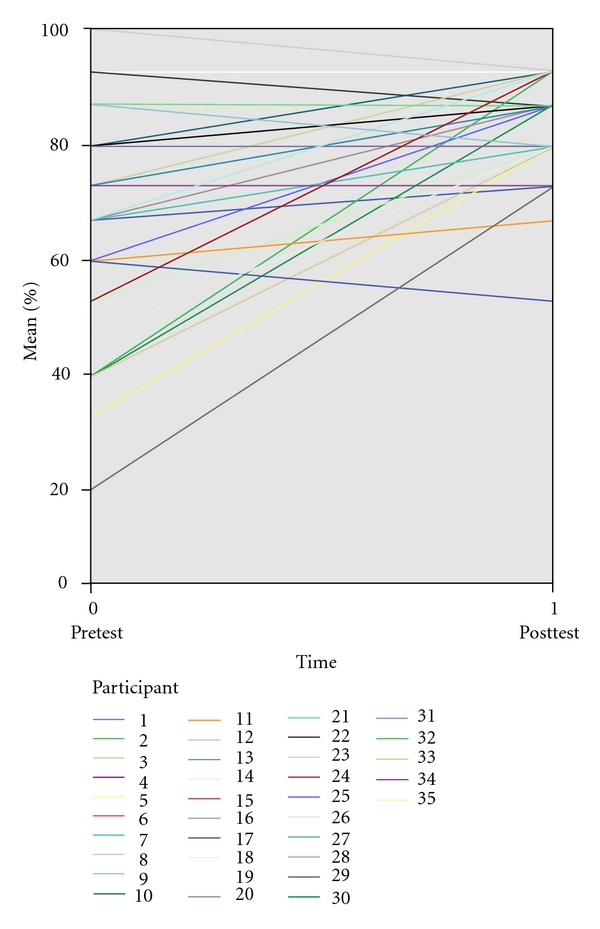
Before and after test plot.

Figure 3 shows a before-and-after plot for all subjects enrolled in the study. 

## 4. Discussion

The use of ultrasound in the ED, in general, appears to be increasing, with greater usage in academic centers than in community hospitals, but evidence from at least one statewide survey suggests that bedside ultrasound is still underutilized despite cost/time efficiency, and quality assurance measures [14, 15]. Among the many uses for ultrasound in the ED, bedside echocardiography appears particularly beneficial, since it is both sensitive and specific for detecting acute myocardial infarction by the presence of regional wall-motion abnormalities. In addition, it does not appear to be appropriate in other settings, because optimal sensitivity requires imperatively that the examination be performed while the patient is experiencing the symptoms of chest pain, which is typically the time of presentation to the ED [1–3, 6, 16–20]. If the study is done during a period without pain, significant coronary artery disease may be missed, because the ischemic wall-motion abnormality may resolve rather quickly [21]. 

 Prior studies have evaluated emergency physicians' ability to obtain images, grade ventricular size and function, identify effusions, and evaluate for signs of ischemic sepsis [5, 8, 11, 22, 23]. Although there are demonstrated benefits of cardiac ultrasound performed at the bedside in the emergency department to evaluate patients with chest pain, to our knowledge, a specific attempt to quantify the proficiency of noncardiologist physicians to identify ischemic or isolated cardiac wall-motion abnormalities has not been previously reported. 

Our study provides evidence that emergency physicians can readily learn how to identify regional wall-motion abnormalities on echocardiograms. Proficiency and further mastery can only progress from there due to potential for high and continuous exposure.

The training module we developed consisted of images taken from readily available online and DVD sources, and could be completed by an instructor in approximately half an hour. Because the pretest and posttest each took approximately 15 minutes, the total time involved for testing, training, and evaluation was only approximately 1 hour. Additional training can only benefit proficiency levels in this practice and the emergency physician will have enough exposure in the acute setting of the ED to further his skill, as chest pain is constantly ranked among the highest complaint seen in the ED. Further study would be useful to determine quality assurance measures as far as continued training and testing.

Many ultrasonographic studies are only relevant to be performed and interpreted at the bedside urgently such as evaluation of pulseless electrical activity. If it is not approved of the EP to perform such exams, the technology is simply wasted. The more definitive picture that an echocardiogram can offer in the setting of PEA or myocardial ischemia can potentially save a great deal of resources and save the patient from unneeded therapy.

Emergency department echocardiography performed in patients with a broad range of risk of myocardial ischemia identifies those at high risk of cardiac events and provides significant incremental diagnostic value when added to baseline clinical, historical, and EKG variables [2]. 

It has been found that formal two-dimensional echocardiography during pain in patients with a nondiagnostic electrocardiogram can readily identify coronary artery disease in the emergency room *and* can accurately rule out an acute myocardial infarction [3]. An echocardiographic study displaying a normal left ventricular wall motion during chest pain is a strong predictor of a nonischemic etiology and, thus, low risk. Wall-motion analysis may also be useful when the EKG displays left bundle branch block or a paced rhythm, where EKG interpretation fails to reveal ischemia [24]. The practice of bedside echocardiogram has the potential to reduce the number of false positive cases of myocardial ischemia, which activate aggressive protocols of medications, admissions, and angiography.

Brief ultrasonographic examination has better diagnostic accuracy at identifying cardiac abnormalities than physical examination in the emergency department setting and provides prognostic data regarding length of hospital stay [18]. 

Few emerging medical technologies have drawn such interest and interdisciplinary controversies as emergency bedside diagnostic ultrasonography [16]. Early resistance to the use of the ultrasound in the ED stemmed from a lack of guidelines specifying appropriate training and indications [25]. A source of further controversy could be encroachment into other medical specialties such as radiology, or in this case, cardiology, that quality within a specialized realm is compromised when a nonspecialist performs the duties. However, EPs are trusted and expected to perform gynecological, ophthalmologic, and a wide breadth of other exams and procedures and to use the instruments of these specialties with proficiency. 

Subsequently, a number of studies, like this one, fed the needed data for guidelines and feasibility of a noncardiologist performed protocol for echocardiography. Briefly, trained emergency personnel of two nurses, one cardiovascular technician, and one paramedic performed a brief screening cardiac ultrasound. Their accuracy of findings were compared to the gold standard findings from a chest pain center, where the exams were performed by an experienced cardiac sonographer or cardiologist. Significant cardiac findings, including left ventricular dysfunction, mitral regurgitation, aortic stenosis, aortic regurgitation, mitral stenosis/prosthesis, aortic root disorders, and pericardial effusion, were reliably found in 22 of 30 patients [18]. This is compelling evidence for the viability of a program for EPs to learn and perform echocardiography usefully.

Our study does come with a series of limitations. This study was not based on a minimum or “passing” requirement for recognizing wall-motion abnormalities. Assigning a cutoff score, however, that is agreed upon by echocardiography, cardiology, and emergency experts would give a more concrete picture of the usefulness of the teaching intervention. In addition, further studies that focus on physicians who “fail” the initial exam could be useful. The number of subjects of the failing population that then passed after the teaching intervention and the amount of remediation of the remaining failures would require to achieve proficiency would give a clearer perspective on the degree of learning curve. We did evaluate physician interpretations as a dichotomous outcome (either the presence or absence of a regional wall-motion abnormality) but limited further analysis of physician's ability to grade severity of regional wall-motion abnormalities on echocardiograms. Future tests should have a passing/failing cutoff as well as a severity grade component. Although it has been suggested that the basic skills of echocardiographic interpretation may be more easily learned than electrocardiogram interpretation, we did not make any attempts to measure or compare physicians' perception of ease of learning. In addition, the testing modules were created from multiple sources, and have not been formally validated for this purpose. Likewise, the small sample size we studied at a single site may limit generalizability to other institutions. Also, we did not evaluate physicians' ability to actually perform cardiac ultrasound and obtain specific images, as has been done by other investigators [23, 24]. Another limitation was that all subjects were given the same 15 cases for pre- and posttests which may have contributed to the consistency of our results and not indicative of the consistency found in true practice or if the cases had been scrambled. Finally, we have not tested for continued retention of knowledge after our initial study. A repeated assessment of the physicians tested at 3 to 6 months would better demonstrate the reliability of EPs to consistently diagnose wall-motion abnormalities and, therefore, reinforcing the practicality of the skill.

## 5. Summary

In conclusion, with only brief training on how to recognize wall-motion abnormalities on echocardiograms, emergency physicians showed significant improvement in their ability to identify wall-motion abnormalities on echocardiograms. The ability of noncardiologists to learn techniques of identification of wall-motion abnormalities suggests that routine incorporation of bedside cardiac ultrasound by emergency physicians may be feasible.
